# Determinants of the Quality of Life in Patients with Drug-Resistant Temporal Lobe Epilepsy: A Comparison of the Results before and after Surgery

**DOI:** 10.3390/brainsci14030241

**Published:** 2024-02-29

**Authors:** Aleksandra Bala, Agnieszka Olejnik, Michał Kułak, Andrzej Rysz, Tomasz Dziedzic, Arkadiusz Nowak, Andrzej Marchel, Przemysław Kunert

**Affiliations:** 1 Faculty of Psychology, University of Warsaw, Stawki 5/7, 00-183 Warsaw, Poland; 2 Department of Neurosurgery, Medical University of Warsaw, Banacha 1a, 02-097 Warsaw, Poland

**Keywords:** temporal lobe epilepsy, epilepsy surgery, quality of life, depression

## Abstract

Drug-resistant temporal lobe epilepsy is associated with a reduction in the quality of life of patients. The aim of this study was to compare the quality of life before and after the surgical treatment of epilepsy and to assess factors that may affect the well-being of patients after surgery. The study involved 168 patients with drug-resistant temporal lobe epilepsy. All of them were examined twice: once before and again one year after surgery. Two questionnaires were used in the study: the Quality of Life in Epilepsy Inventory-Patient-Weighted and Hospital Anxiety and Depression Scale and one that collected data on selected demographic and clinical variables. The results showed that patients scored significantly higher in quality of life and lower in depression and anxiety after surgery; however, this only applied to patients with a good outcome of treatment (Engel Class I and Class II). Patients with an unfavorable outcome of surgical treatment (Engel Class III and Class IV) achieved significantly worse results in all examined variables. Correlational analysis showed a relationship between select aspects of quality of life and the level of depression and anxiety, as well as the frequency of seizures and age at epilepsy onset. There was no significant relationship with age, sex, education, or number of prescribed antiepileptic drugs. The study confirms the significant relationship between the quality of life and the effectiveness of surgical treatment, indicating the relationship between patients’ well-being and selected clinical indicators.

## 1. Introduction

Epilepsy is one of the most common chronic neurological diseases and is associated with a stigma and lower quality of life (QoL) [1,2,3]. The prevalence of active epilepsy worldwide is 6.38 per 1000 persons and does not vary by sex or age, yet the lifetime prevalence and incidence rates were significantly higher in the countries with low to middle income [4]. Epilepsy can be described as repetitive and aberrant interruptions of brain function, which is recognized as epileptic seizures [5]. One of the most common epilepsies is temporal lobe epilepsy (TLE), which is estimated to comprise 30% of all epilepsies and 60% of focal epilepsies, making TLE the most frequent culprit of focal seizures [6]. Patients with TLE tend to suffer from mood disorders (24–74%), depression (30%), anxiety (10–25%), and psychiatric disorders (10–20%) [7]. Moreover, they may experience various cognitive impairments (regarding, e.g., memory, executive functions, language, or theory of mind) [8,9,10,11,12]. Psychiatric comorbidities combined with frequent seizures, stigma, and possible cognitive impairments may be some of the reasons for lower QoL in patients with TLE [2,3,7]. The most effective treatment method used in drug-resistant TLE is surgery. Research conducted by Tanriverdi and co-authors [13] proved that the rates of seizure freedom after resective TLE surgery in 6 months, 2 years, and 12 years were 82.5, 76.2, and 70.8%, respectively. In general, surgery can help to prevent epileptic seizures in about 35–80% of all patients [14].

Previous studies have explored and identified key factors influencing QoL (referred to as health-related QoL or HRQoL) of individuals living with epilepsy. Findings consistently highlight factors such as depression, anxiety, seizure frequency, epilepsy type, age of epilepsy onset, and sleep disturbances, as well as social and personal support, as significant determinants impacting QoL in patients with epilepsy [15,16,17,18,19,20,21,22,23,24]. Some researchers claim that QoL may also be influenced by surgical treatment [25,26], but data on the impact of individual factors that would be responsible for the change in the well-being of patients after surgery are inconclusive due to the heterogeneity of the studied groups, the lack of repeatable measurement methods, and the heterogeneity of the studied variables. Available data suggest that the factors that may be associated with a patient’s psychological outcome after surgery include seizure-free status, time of seizure freedom, side effects of surgery, intake of epileptic drugs, side effects of those drugs, employment, and the presence of aura [27,28]. However, due to the inconsistency of the conclusions, further research is needed in this area.

The aim of the present study was to assess the quality of life in patients with temporal lobe epilepsy before and after surgery and to search for the clinical and demographical factors associated with the well-being of patients after surgery.

## 2. Methods

### 2.1. Participants

Patients whose diagnosis suggested the possibility of surgical treatment of drug-resistant temporal lobe epilepsy, at the Department of Neurosurgery of the Medical University of Warsaw, were invited to participate in the study. The inclusion criteria was the confirmed diagnosis of drug-resistant TLE on the basis of the phenomenology of seizures and with the use of video-EEG (video-electroencephalography), MRI (magnetic resonance imaging), and PET (positron emission tomography). All patients in the follow-up study underwent anterior temporal lobectomy surgery.

**Figure 1:**
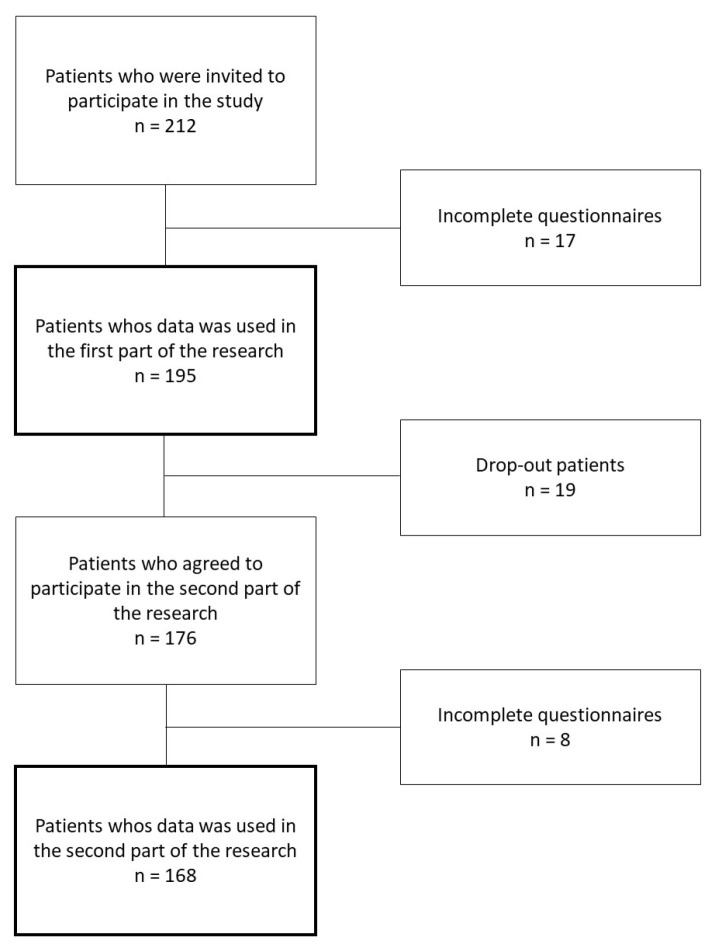
Flowchart representing the sample size process.

In the first stage of the study, we obtained data from one hundred and ninety-five patients, but due to the reasons described below, only one hundred and sixty-eight subjects fully participated in the second stage, thus, only these subjects were included in the further analyses. Details of patient selection can be found in the flowchart below (Figure 1).

During the first assessment, 97 women and 71 men took part in the study. Patients were on average 32.2 years old (SD = 11.9) in the first study and during the second study, patients were on average 33.4 years old (SD = 12.2). The mean duration of formal education was 13.1 years (SD = 4.3). The onset of epilepsy on average was at the age of 12.5 years (SD = 11.6) and the average number of attacks per month was 5.45 (SD = 3.4) before surgery and 0.9 (SD = 2.1) after surgery.

### 2.2. Procedure

The study was conducted twice: a few days before the surgery and approximately 12 months after the surgery. In both cases, the patients were examined by a trained psychologist in the hospital: the first time was during the pre-operative diagnosis and the second time was during a follow-up visit. After a standard clinical interview and neuropsychological screening, patients were asked to complete two questionnaires. They completed them sitting in a well-lit, quiet room and had no time limit. They were informed that there were no right or wrong answers and that the researchers only wanted to know the patient’s true opinion about his/her well-being. Information on the frequency and type of seizures was obtained from seizure diaries kept by patients and their families. All procedures were carried out in accordance with the Declaration of Helsinki and were approved by the ethics committee of the Faculty of Psychology at the University of Warsaw.

### 2.3. Assessment Tools

For the assessment of patients’ psychological status, we used two questionnaires:

(a) Quality of Life in Epilepsy Inventory-Patient-Weighted (QoLIE-31-P) [29] is a widely utilized questionnaire designed to assess the quality of life in adults with epilepsy. It comprises seven distinct scales that each address different aspects of an individual’s life: Emotional Well-Being, Social Functioning, Energy/Fatigue, Cognitive Functioning, Seizure Worry, Medication Effects, and Overall QoL. The cumulative scores from these seven scales provide an estimation of the overall QoL. Furthermore, the questionnaire includes two additional scales: the distress scale and an unscored overall health-level scale, both of which allow for a comprehensive evaluation of an individual’s well-being. Each subtest of the QoLIE-31-P allows respondents to accrue between 0 to 100 points, with higher scores indicating a better QoL. It is noteworthy that the authors of this study employed a validated Polish version of the QoLIE-31-P questionnaire.
(b) The Hospital Anxiety and Depression Scale (HADS) [30] is a self-reporting questionnaire designed to assess and measure symptoms of anxiety and depression in individuals who are receiving medical treatment but do not have a formal diagnosis of a psychiatric disorder. The HADS is widely used in healthcare settings to screen for and evaluate the emotional well-being of patients, particularly those with physical health conditions. It consists of 14 questions, with 7 questions dedicated to assessing symptoms of anxiety and 7 questions for assessing symptoms of depression. These questions are designed to be simple and straightforward, making them easily understandable for a wide range of individuals. For each section (anxiety and depression), a score of 0–7 is considered “normal” or indicative of no or minimal symptoms, scores between 8 and 10 suggest the presence of mild symptoms, scores between 11 and 14 are indicative of moderate symptoms, and scores above 14 may indicate severe symptoms.

### 2.4. Statistical Analyses

Statistical analyses were performed using the Statistical Product and Service Solutions (SPSS) version 29.0 software (IBM Corp., Armonk, NY, USA) for Windows. The Shapiro–Wilk test showed that the data followed a normal distribution and that it was possible to use the parametric tests, as other requirements were also met. The comparisons between patient results obtained before and after surgery were conducted using the Student’s *t*-test for dependent samples. A one-way ANOVA was used to investigate the differences between three groups: (a) before the surgery, (b) after the surgery with a favorable outcome, and (c) after the surgery with an unfavorable outcome. To maximize the reliability of the results, the Scheffé test was used for post hoc comparisons. Pearson’s r coefficient was used in the correlation analysis of the QoLIE-31-P scores with the HADS and clinical variables, such as age, sex, education, number of seizures per month, age at epilepsy onset, number of prescribed antiepileptic drugs. The η coefficient was used to assess the correlation between nominal and quantitative variables. The chi^2^ test was used to compare frequency distributions of categorical variables. Multiple linear regression analysis was performed to determine the impact of the clinical variables on the quality of life. Results were considered significant when *p*  < 0.05.

## 3. Results

The results obtained from patients before and after the surgery were compared. The details are presented in Table 1.

As expected, patients with epilepsy achieved higher results in all QOLIE-31-P subscales and lower scores of anxiety and depression in the HADS after surgery than before.

Due to the heterogeneity of the post-operative group in terms of the results of surgical treatment, it was decided to further divide this group into two subgroups in regard to their clinical outcome to better understand the mechanisms regulating the QoL in patients with epilepsy. Post-operative seizure outcome was graded according to the Engel Epilepsy Surgery Outcome Scale [31]. A group of patients with a good and very good treatment outcome (Engel Class I and Engel Class II) and a group with an unfavorable treatment outcome (Engel Class III and Engel Class IV) were identified. The demographic and clinical details of both groups are shown in Table 2.

The comparison between groups showed that patients with a good surgical outcome had significantly less seizures (t = 1.2, *p* < 0.001), and significantly less often had a diagnosis of hippocampal sclerosis associated with focal cortical dysplasia (HS-FCD) (chi^2^ = 15.02; *p* < 0.01). They had also a slightly higher age at epilepsy onset; however, this result was only a statistical trend (t = 0.4, *p* = 0.053). The other indicators of sex, age, and years of education did not differ between groups.

### 3.1. Differences in QoL and Emotional State in Patients with Good and Unfavorable Treatment Outcomes

Next, the results of particular subscales of the QoL questionnaire and the HADS were compared. First, we conducted a t-Student test to retrospectively check whether the group of people with a good surgical outcome differed pre-operatively from the group of people with unfavorable treatment results in terms of mood and quality of life. Analyses showed that there were no significant differences in the results of both questionnaires. Therefore, further comparisons were performed using the pre-operative data of the entire group.

The one-way ANOVA showed significant differences between the studied groups in every subtest of the QOLIE-31-P questionnaire, namely in Distress (F(2.333) = 239.4, *p* < 0.001). The results of the other scales were as follows: Seizure Worry was F(2.333) = 256.2, *p* < 0.001; Medication Effect was F(2.333) = 186.4, *p* < 0.01; Cognitive Functioning was F(2.333) = 325.9, *p* < 0.05; Emotional Well-Being was F(2.333) = 212.1, *p* < 0.05; Energy/Fatigue was F(2.333) = 316.4, *p* < 0.005, and Overall Score was F(2.333) = 347.6, *p* < 0.01. Differences were also present in the HADS scores in Depression (F(2.333) = 24.7, *p* < 0.05) and Anxiety (F(2.333) = 19.9, *p* < 0.05).

**Figure 2:**
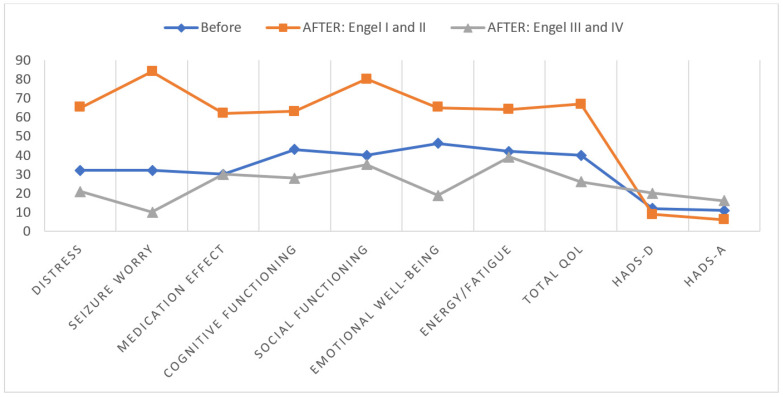
Between-group comparison of quality-of-life questionnaire subscales. Legend: The numbers on the ordinate indicate the level of quality of life measured using the QoLIE-31-P questionnaire, with a range of results from 0 to 100; HADS-A—anxiety subscale of Hospital Anxiety and Depression Scale; HADS-D—depression subscale of Hospital Anxiety and Depression Scale.

Post hoc analyses revealed that patients with a good surgical outcome (Engel Class I and Class II) gained significantly higher scores in all subscales of the QoLE-31-P compared to before surgery and patients with an unfavorable outcome (Engel Class III and Class IV): Energy (*p* < 0.05), Emotional Well-Being (*p* < 0.05), Social Functioning (*p* < 0.05), Cognitive Functioning (*p* < 0.05), Medication Effect (*p* < 0.05), Seizure Worry (*p* < 0.01), and Distress (*p* < 0.01). The level of symptoms of depression and anxiety in this group was slightly lower than before surgery, but the results were not statistically significant (*p* > 0.05). Patients from the unfavorable-outcome group who still had seizures (the same number or slightly less—Engel Class III and Class IV) declared a lower QoL in terms of Emotional Well-Being (*p* < 0.001), Cognitive Functioning (*p* < 0.05), Seizure Worry (*p* < 0.01), and Distress (*p* < 0.01) than before surgery. They had also significantly higher rates of anxiety (*p* < 0.05) and depression (*p* < 0.01) than the group with a favorable outcome and before surgery. To increase clarity, all of the described results are presented in Figure 2.

### 3.2. Correlation between QoL and Other Variables

Correlational analyses were performed for all post-operative patients and showed significant relationships between particular aspects of QoL and other clinical variables. The described results are presented in Table 3.

Most aspects of QoL were negatively correlated with the frequency of seizures and the severity of depressive symptoms. Moreover, people who had an onset of epilepsy at a younger age had a higher QoL in some aspects than those who had epilepsy onset at a later age. Furthermore, some correlations were found between a few subscales of QoL and the level of anxiety measured with the HADS. Analyses covered other indicators such as age, sex, education, side of epileptic focus, extent of resection, histopathological result, mono/polytherapy or number of prescribed antiepileptic drugs, but none of them were significantly associated with QoL and were not included in Table 3.

Next, a multiple linear regression was calculated to predict the total quality of life based on selected clinical variables. A significant regression equation was found F(4,163) = 27.14; *p* < 0.05, with an R^2^ of 0.39. Total QoL decreased by 1.71 points for each seizure per month, 2.46 points for each year of age at epilepsy onset, 3.23 points for each point of HADS-D (depression subscale of HADS) and 2.73 points for each point of HADS-A (anxiety subscale of HADS). All four variables were significant predictors of the total quality of life.

## 4. Discussion

The present study aimed to explore the changes in quality of life in patients diagnosed with drug-resistant temporal lobe epilepsy before and after surgical treatment, as well as the determinants of QoL in the post-surgical group. Previous studies proved that TLE, which accounts for a substantial proportion of epilepsy cases [32], significantly impacts various facets of patients’ lives including mental health [33], cognitive functions [11,34], and social interactions [9,35]. Surgery has been identified as one of the most effective treatments for drug-resistant TLE [32] and was assessed in our study for its impact on patients’ QoL.

The observed improvements in various domains of QoL, as measured by the QoLIE-31-P questionnaire, align with prior research indicating the positive impact of surgery on patients’ well-being [25]. In particular, many patients exhibited higher scores across emotional, social, cognitive, medication-related, and seizure-related aspects after surgery, indicating an overall enhanced QoL. Moreover, the notable decrease in anxiety and depression symptoms, which was assessed using the HADS questionnaire, underscores the positive effects of surgical intervention on patients’ emotional health. This improvement is worth emphasizing, given the high prevalence of mood disorders among individuals with TLE [36,37] that significantly affects their QoL [25,38]. The obtained results are in line with previous studies in this field [39], but there are other studies showing no improvement or worsening in this area in some patients [40,41].

Dividing the patients into two groups allowed for a better insight into the nature of the post-surgical changes in patients’ well-being. Patients with favorable outcomes (Engel Class I and II) demonstrated substantial improvements in seizure occurrence and reported higher QoL scores across all QoLIE-31-P subscales compared to their pre-surgery status and to patients with unfavorable outcomes (Engel Class III and IV). Conversely, patients with ongoing seizures after surgery displayed persistently lower QoL, particularly in emotional well-being, cognitive functioning, and distress. Additionally, this group reported higher levels of depression than both their pre-surgery status and the group with favorable outcomes. These findings emphasize the critical role of seizure control in influencing post-surgery QoL. Patients experiencing fewer seizures after surgery exhibited superior QoL across multiple dimensions, highlighting the significance of successful seizure management in enhancing overall well-being.

Correlation analyses showed the significant impact of seizure frequency, age of epilepsy onset, and severity of depressive and anxiety symptoms on various aspects of patients’ QoL. Higher seizure frequency and greater severity of depression were correlated with lower QoL in most subscales, while an earlier onset of epilepsy exhibited associations with better QoL in certain domains. Furthermore, relationships between QoL subscales and anxiety levels measured by the HADS were observed, emphasizing the multifaceted interplay of these variables in determining patients’ well-being post-surgery. Multiple linear regression analysis showed that the severity of depression and anxiety, the frequency of epileptic seizures and the age at onset of epilepsy were significant predictors of overall quality of life. These results suggest that reducing seizure frequency and addressing depressive symptoms could significantly contribute to enhancing QoL in patients with temporal lobe epilepsy. However, the relationship between post-operative quality of life and individual clinical variables is not clear. The literature contains studies that show a relationship between QoL and the frequency of seizures [42], age at epilepsy onset [23], and the severity of symptoms of depression and anxiety [42,43,44]; however, there are also studies in which this relationship was not demonstrated (age at epilepsy onset: [43,45,46]; seizure frequency: [43]. The present study did not show the association between post-surgical QoL and other analyzed demographical or clinical variables (which was similar to other studies) such as sex [46], age [47], education level [46], or number of prescribed antiepileptic drugs [43].

Despite the high value of this research due to the important conclusions that it provided, the study was not free from limitations. First, regarding the follow-up duration, the study assessed the quality-of-life changes 12 months post-surgery, but multiple and longer-term follow-ups could provide a more comprehensive understanding of sustained QoL improvements or potential fluctuations over an extended period, offering insights into the long-term efficacy of surgical intervention. While the study examined various clinical factors (seizure frequency, age of epilepsy onset, etc.) and their associations with QoL, other potentially relevant variables (e.g., socioeconomic status and social support) were not included, which might contribute significantly to post-surgery outcomes. In addition, this was a single-center study, and the research was conducted in a specific medical setting, which might not fully represent the diverse demographic and clinical profiles present in other healthcare environments or geographic regions. Finally, including patients with extra-temporal epilepsy would allow us to determine whether the examined properties are specific only to patients with TLE or whether they can be generalized to the entire population of patients with drug-resistant epilepsy. Addressing these limitations in future studies could offer a more comprehensive understanding of the multifaceted determinants influencing QoL in patients with drug-resistant TLE who are undergoing surgical interventions, ultimately guiding more targeted and effective interventions to enhance patient well-being.

## 5. Conclusions

In conclusion, our study reaffirms the substantial association between surgical intervention outcomes and the quality of life of patients grappling with drug-resistant temporal lobe epilepsy. The observed improvements in various domains of QoL post-surgery alongside reduced symptoms of anxiety and depression underscore the holistic benefits of successful surgical intervention in enhancing patients’ emotional and social well-being. The division of patients based on treatment outcomes highlights the pivotal role of seizure control in determining post-surgery quality of life, emphasizing the significance of effective seizure management strategies. The findings also stress the multifaceted nature of factors associated with post-surgery QoL and emphasize the importance of tailored interventions to optimize the well-being of individuals living with drug-resistant temporal lobe epilepsy.

## Figures and Tables

**Table 1:** Comparison of scores obtained from patients before and after epilepsy surgery treatment.

		Before Surgery M (SD)	After Surgery M (SD)	Statistics	*p* Value	Cohen’s d
QOLIE-31-P	Distress	34.3 (8.8)	44.3 (7.4)	t = 1.23	*p* = 0.007	0.61
	Seizures Worry	35.7 (7.5)	47.1 (6.9)	t = 2.01	*p* = 0.002	0.72
	Medication Effects	33.2 (6.9)	48.4 (5.3)	t = 3.98	*p* = 0.004	0.65
	Cognitive Functioning	45.1 (7.1)	47.8 (8.3)	t = 2.27	*p* = 0.039	0.34
	Social Functioning	44.5 (5.2)	54.6 (4.6)	t = 2.63	*p* = 0.009	0.31
	Emotional Well-Being	48.4 (9.5)	53.9 (6.9)	t = 3.12	*p* = 0.021	0.25
	Energy/Fatigue	45.2 (3.6)	53.2 (3.9)	t = 2.45	*p* = 0.046	0.35
	Total	43.1 (7.9)	49.4 (5.5)	t = 1.99	*p* = 0.008	0.48
HADS	Depression	15.1 (9.6)	9.7 (7.3)	t = 3.12	*p* = 0.045	0.46
	Anxiety	13.6 (8.1)	8.9 (6.9)	t = 2.57	*p* = 0.039	0.51

**Table 2:** Demographic and clinical structure of post-operative groups divided according to surgical outcome.

	Post-Operative Group: Engel I and II n = 136	Post-Operative Group: Engel III and IV n = 32	*p* Value
Age, M (SD)	32.7 (13.6)	34.3 (12.1)	*p* > 0.05 *
Sex, M/F	57/79	13/19	*p* > 0.05 **
Years of formal education, M (SD)	13.3 (3.1)	13.2 (4.3)	*p* > 0.05 *
Epilepsy Focus, R/L	70/66	17/15	*p* > 0.05 **
Age at epilepsy onset, M (SD)	13.4 (9.1)	12.1 (8.8)	*p* = 0.053 *
Seizures per month, M (SD)	0.31 (0.1)	6.41 (5.7)	*p* < 0.001 *
Extend of ATL resection, cm from temporal pole, M (SD)	3.92 (1.4)	4.01 (1.2)	*p* > 0.05 *
Histopathology: HS/HS-FCD	89/47	12/20	*p* < 0.01 **
Treatment status: mono-/polytherapy	5/131	1/28	*p* > 0.05 **

Legend: *—t-Student test; **—chi^2^ test; M—mean; SD—standard deviation; ATL—anterior temporal lobectomy; HS—hippocampal sclerosis, HS-FCD—hippocampal sclerosis associated with focal cortical dysplasia; significant scores are in bold.

**Table 3:** Correlation analysis of QoL subscales and selected clinical indicators in the post-surgical group.

	HADS-A	HADS-D	Seizures/Month	Epilepsy Onset
**QoL:** Distress	r = −0.44; *p* < 0.05	r = −0.48; *p* < 0.05	r = −0.63; *p* < 0.001	
**QoL:** Seizures Worry	r = −0.53; *p* < 0.01	r = −0.46; *p* < 0.05	r = −0.52; *p* < 0.01	r = −0.43; *p* < 0.01
**QoL:** Medication Effects			r = −0.38; *p* < 0.05	
**QoL:** Cognitive Functioning		r = −0.51; *p* < 0.05	r = −0.41; *p* < 0.05	r = 0.35; *p* < 0.05
**QoL:** Social Functioning	r = −0.66; *p* < 0.05	r = −0.42; *p* < 0.05	r = −0.42; *p* < 0.05	r = −0.62; *p* < 0.01
**QoL:** Emotional Well-Being	r = −0.39; *p* < 0.05	r = −0.46; *p* < 0.01	r = −0.71; *p* < 0.01	
**QoL:** Energy/Fatigue		r = −0.72; *p* < 0.01	r = −0.33; *p* < 0.05	
**Total QoL**	r = −0.31; *p* < 0.05	r = −0.52; *p* < 0.05	r = −0.44; *p* < 0.05	r = −0.55; *p* < 0.01

Legend: r—Pearson’s coefficient; HADS-A—anxiety subscale of Hospital Anxiety and Depression Scale; HADS-D—depression subscale of Hospital Anxiety and Depression Scale.

## Data Availability

The data are not publicly available due to the confidentiality issues but are available on reasonable request from the corresponding author.
